# The Orphans’ Home

**Published:** 1867-06

**Authors:** 


### THE ORPHANS' HOME.

The masses have a settled feeling of hostility, and even bitterness toward the rich, as if they were their hereditary enemies, and yet in no large city of the world is so much done for suffering humanity as by the rich, the fashionable, and the aristocratic. It is easy for them to give money and they do it; but they do more; they give their countenance, their time, and their personal efforts for the purpose of raising means to help the poor, the needy, the sick and the friendless. It is from their purses funds come, and from their influence and individual efforts plans are carried out, which eventuate the noblest charities of our time, the colleges, the homes, the asylums, the hospitals, which nurse the sick, which care for the insane, which shelter the unfortunate, which feed the hungry, and guard and guide and cherish the forsaken and the motherless; and it is to those who keep the "fatherless" that the inspired page awards the meed of citizenship in Heaven.

The "Orphans' Home" of the city of New York, is one of the most humane of all charities, and to raise funds for its support, the splendid mansion of Dr. Ward of 47th St., was opened on a May evening, as many times before, for an amateur musical entertainment. The tickets were promptly disposed of at a high price, as is always the case when anything of the kind is to come off in 47th St., because the public have got to know that things are sure to be well done there, that there will not be a vacant chair, and that the highest of the high will be present with their refinements, and cultivated and chastened tastes. It is only the "canaille" who dress violently; only the "new rich" who crowd their parlors with furniture, and whose upholstery and hangings are resplendent with red and yellow and blue. But there is nothing of all this in "the house of the city," as an elegantly dressed woman very appropriately expressed herself in our hearing, of the dwelling of which we are writing, with its hundred feet front, when the most ambitious among us can only boast of twenty-five, with here and there a thirty. This mansion is regarded by those who have the "entree" of the most magnificent residences in New York, as by far the most unexceptionably furnished dwelling in the city. There is nothing tawdry, no tinsel, no imitation; everything is real, chaste, substantial and consonant with its uses and surroundings. The music room where the entertainment was held is of the entire length of the building and of proportioned width, and is lighted from a dome in the centre by the sun in the day, and by gas at night. All the performers in the evening were volunteers for the good cause, and came from the ranks of our most aristocratic families; the music itself was amateur and composed for the occasion; all performed their parts to the admiration and frequent plaudits of the auditors. Miss E. A. as the old negress, deserves high praise, and Miss Freeman with her laughing eyes and womanly beauty and birdlike voice, fairly carried the whole company away; and as Gen. M. enthusiastically said of her performance, "Dr., it is more than excellent." The whole entertainment was a perfect success, and although the whole street was blocked up with splendid equipages, and collisions and confusions and mistakes seemed inevitable, yet nothing occurred to mar the general enjoyment, for "Brown" was there.

